# Community-based primary care approaches to supporting families of children with developmental disabilities: Experts’ perspectives using the capabilities framework

**DOI:** 10.4102/safp.v67i1.6217

**Published:** 2025-12-17

**Authors:** Lumka Magidigidi-Mathiso, Jose Frantz, Gerard Filies

**Affiliations:** 1 Centre for Interdisciplinary Studies of Children, Families and Society, Faculty of Community and Health Sciences, University of the Western Cape, Cape Town, South Africa; 2 Department of Physiotherapy, Faculty of Community and Health Sciences, University of the Western Cape, Cape Town, South Africa

**Keywords:** developmental disabilities, interdisciplinary approach, experts, family support, capabilities framework, children, community-based, primary healthcare

## Abstract

**Background:**

Families raising children with developmental disabilities face complex, interconnected challenges requiring coordinated support across multiple professional domains. While interdisciplinary collaboration is widely endorsed in policy and practice guidelines, significant knowledge gaps exist regarding how healthcare professionals operationalise these collaborative approaches in real-world settings.

**Methods:**

A qualitative study was conducted using individual semi-structured interviews with 12 experts representing diverse disciplines. The study was grounded in the Capabilities Approach as both a theoretical lens and a methodological framework. Data were analysed using Braun and Clarke’s six-step thematic analysis, with the Capabilities Approach framework informing each analytical phase.

**Results:**

Five major themes emerged from the capability-guided analysis: facilitating emotional transformation, building system navigation competence, creating inclusive participation opportunities, strengthening family functioning and fostering adaptive identity development. Across all themes, participants consistently emphasised three critical mechanisms for effective interdisciplinary support: coordinated care delivery as capability enhancement, comprehensive emotional support as capability development and whole-family capability strengthening interventions.

**Conclusion:**

The findings highlight the significance of interdisciplinary approaches informed by the Capabilities Approach in providing comprehensive support for parents of children with developmental disabilities. Rather than traditional deficit-focused models, participants systematically described how they enhance family capabilities by functioning as capability facilitators who orchestrate conversion factors, build emotional capabilities while respecting family agency and create environmental modifications that expand family possibilities.

**Contribution:**

This study contributes a novel capability-focused framework for understanding interdisciplinary collaboration in developmental disability support, moving beyond traditional service coordination models to emphasise systematic capability enhancement approaches that build sustainable family strengths across multiple domains simultaneously.

## Introduction

### Social value

Developmental disabilities affect approximately one in six children aged 3–17 years in the United States and represent a diverse group of conditions.^1^ Raising a child with developmental disabilities presents unique and multifaceted challenges for families, requiring extensive coordination across healthcare, educational and community support systems. Parents of children with developmental disabilities often report higher levels of stress, financial strain and social isolation compared to parents of typically developing children.^2,3^

The care and support of children with developmental disabilities have evolved significantly over recent decades, moving from primarily medical models towards more holistic, family-centred approaches that acknowledge the interconnected nature of child and family wellbeing.^4,5^ However, fragmentation of services remains a persistent challenge, with families often reporting difficulties navigating complex and disconnected support systems.^6,7^

### Scientific value

Interdisciplinary approaches involve the integration of expertise from diverse professional disciplines, including medicine, nursing, social work, psychology, education and therapy services. These approaches recognise that no single discipline possesses the comprehensive knowledge and skills required to address the multidimensional needs of children with developmental disabilities and their families.^8,9^

Despite evidence supporting the theoretical benefits of interdisciplinary approaches, there remains a limited understanding of how experts conceptualise and implement these collaborative models in practice. This gap is particularly concerning given that effective interdisciplinary collaboration not only requires structural changes but also shifts in professional perspectives, communication patterns and service delivery approaches.^10,11,12^

This article builds upon foundational work from a broader PhD research programme that aims to develop guidelines for enhancing the abilities of parents with children who have developmental disabilities. Utilising an interdisciplinary approach, an article derived from this PhD research examined ways to strengthen parental capabilities and investigated the role of interdisciplinary support in improving both parental well-being and effectiveness in caring for children with developmental disabilities.^13^

### Conceptual framework

This study was fundamentally grounded in the Capabilities Approach^14,15^ as both a theoretical lens and methodological framework that guided every stage of the research process from conceptualisation through analysis. The Capabilities Approach, developed by Sen and Nussbaum, focuses on what people can do and be, rather than on the resources they possess or their subjective well-being. This framework emphasises human dignity, individual agency and the importance of social arrangements in enabling people to achieve their full potential.

Rather than applying capability theory post hoc to interpret findings, the framework actively shaped research design, data collection instruments, analytical processes and knowledge construction. This approach allowed for the examination of how professional practices influence capability development across multiple domains simultaneously.

### Aim and objectives

This study aimed to explore expert perspectives on community-based primary care approaches for supporting families of children with developmental disabilities using the Capabilities Framework. The specific objectives were to understand how interdisciplinary experts conceptualise comprehensive family support, identify key mechanisms through which collaborative approaches enhance family capabilities, examine how professionals address conversion factors that enable or constrain family functioning and develop a capability-focused framework for interdisciplinary collaboration in developmental disability support.

## Research methods and design

### Study design

This study employed a qualitative research design using semi-structured interviews to collect rich, detailed data on the experiences and perspectives of experts who work directly with children with developmental disabilities and their families. A qualitative approach was selected to capture the nuanced perspectives and complex experiences that quantitative methods might overlook, particularly regarding interdisciplinary collaboration and support strategies.

### Setting

The study was conducted in the Western Cape Province of South Africa, encompassing urban, peri-urban and rural communities. Participants worked across diverse settings, including public hospitals, community health centres, non-governmental organisations, private practices and community-based organisations. This geographic and institutional diversity provided comprehensive insights into how interdisciplinary approaches function across different resource contexts and service delivery models.

### Study population and sampling strategy

Participants were recruited through purposive sampling to ensure representation across multiple disciplines involved in supporting children with developmental disabilities and their families. Participant selection criteria were capability-informed, prioritising professionals who worked across multiple capability domains rather than single-discipline experts. This ensured data collection would capture the systemic, interdisciplinary approaches that capability theory suggests are necessary for comprehensive human development support.

Inclusion criteria included professionals with at least 3 years of experience working directly with children with developmental disabilities and their families, current involvement in interdisciplinary or multidisciplinary care teams and experience across multiple professional settings or with diverse family populations. Exclusion criteria included professionals working exclusively in administrative roles without direct family contact and those unable to participate in interviews conducted in English.

Recruitment strategies included (1) initial contacts through established professional associations for healthcare workers, social workers and disability service organisations, (2) snowball sampling where participants recommended colleagues from different disciplines and (3) direct organisational outreach to non-governmental organizations (NGOs), hospitals, community health centres and rehabilitation facilities.

A sample of 12 participants was determined appropriate for this study based on data saturation, which was reached after the 10th interview with no new themes emerging in the final two interviews, adequate disciplinary representation across five professional disciplines with 2–3 participants per discipline, alignment with qualitative research norms where samples of 8–15 participants are considered adequate for achieving rich, detailed insights in phenomenological and thematic analysis studies^16,17^ and practical limitations of time and resources for this exploratory study. To enhance trustworthiness, we adhered to Lincoln and Guba’s criteria: credibility through prolonged engagement with participants and member checking of preliminary themes, transferability through thick description of context and participants, dependability through detailed audit trails of analytical decisions and confirmability through reflexive journaling and peer debriefing with the research team.

### Data collection

Individual semi-structured interviews were conducted, with each interview lasting approximately 60–90 min and conducted either in-person at the participant’s workplace or via a secure voice call conferencing platform, based on participant preference.

The interview guide was systematically constructed using Nussbaum’s 10 central capabilities as organising principles, with questions designed to examine how professional practices influence each capability domain. The interview domains included Life and Bodily Health Capabilities exploring how professionals assess and support families’ access to healthcare and basic needs, Emotions and Affiliation Capabilities examining how professionals support families through the emotional journey following diagnosis, Practical Reason and Control over Environment investigating how professionals build families’ decision-making skills and advocacy capabilities and Play and Other Species-Typical Activities exploring how professionals ensure children can participate in age-appropriate recreational and educational activities.

The Capabilities Approach framework specifically guided data collection through capability-focused questions that explored how different support approaches enable or constrain the development of key capabilities in both children and families, examination of how social, institutional and environmental factors influence family well-being and child development, strengths-based inquiry that explored how experts identify and build upon family strengths and capabilities rather than focusing solely on deficits and contextual understanding of how broader systems and structures impact individual and family capabilities.

### Data analysis

All interviews were audio-recorded with participants’ consent and transcribed verbatim by a professional transcription service, with transcripts checked for accuracy against the original recordings and anonymised before analysis.

Data were analysed using Braun and Clarke’s six-step thematic analysis approach,^16^ with the Capabilities Approach framework informing each analytical phase. The six steps included familiarisation with the data through repeated reading of transcripts and initial note-taking, generating initial codes by systematically coding interesting features across the entire dataset, searching for themes by collating codes into potential themes and gathering relevant data, reviewing themes to ensure they worked in relation to coded extracts and the entire dataset, defining and naming themes to refine the specifics of each theme and producing the report by selecting compelling extract examples and relating the analysis back to the research questions and literature.

### Ethical considerations

Ethical approval was obtained from the University of the Western Cape Biomedical Research Ethics Committee on 17 March 2023 prior to data collection. The ethical clearance number is BM23/1/10. All participants provided written informed consent after receiving detailed information about the study purpose, procedures, potential risks and benefits and their rights as research participants.

The lead researcher maintained a reflexive journal throughout data collection and analysis to document potential biases stemming from her dual role as researcher and clinician working with families of children with disabilities. Regular debriefing sessions with the research team provided external perspectives to challenge interpretations and ensure findings emerged from the data rather than researcher assumptions. We also considered the potential impact on participants, recognising that reflecting on their professional practices could evoke both positive insights and challenges to their current approaches. All participants were offered access to study findings and debriefing opportunities.

## Results

**FIGURE 1:**
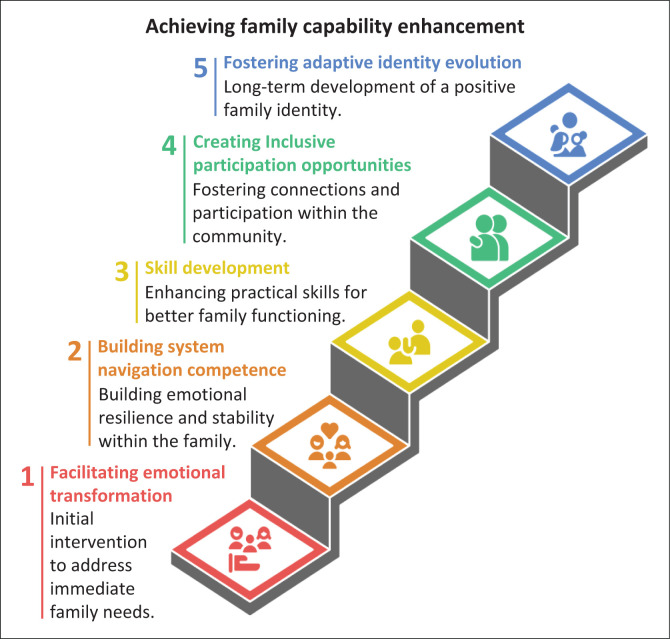
Capability enhancement process.

The thematic analysis revealed five interconnected themes that illuminate how collaborative approaches systematically enhance family capabilities when supporting children with developmental disabilities. Table 1 presents the demographic characteristics of the 12 participants, showing representation across five professional disciplines (medical doctors, nurses, social workers, home-based care workers and NGO founders) with experience ranging from 3 to 15+ years. Table 2 maps each theme to specific capability dimensions from Nussbaum’s framework, demonstrating how the themes systematically address multiple interconnected capabilities. These themes demonstrate the complex processes through which interdisciplinary participants move beyond traditional service delivery models to function as capability facilitators. Figure 1 illustrates the capability enhancement process and the connections between the themes, showing how the five themes work together cyclically to support families, with emotional transformation and adaptive identity development serving as ongoing processes that interact with the other capability-building mechanisms.

**TABLE 1:** Participant demographics (*N* = 12).

Participant ID	Professional role	Years of experience	Gender	Age (years)	Work setting
P1	Medical doctor	15	Female	40–49	Public hospital
P2	Nurse	8	Female	30–39	Community health centre
P3	Social worker	12	Male	35–44	NGO
P4	Home-based care worker	6	Female	25–34	Community organisation
P5	NGO founder	20	Female	50–59	Non-profit organisation
P6	Medical doctor	22	Male	45–54	Private practice
P7	Nurse	10	Female	30–39	Paediatric ward
P8	Social worker	14	Female	40–49	Family services
P9	Home-based care worker	4	Male	20–29	Community outreach
P10	NGO founder	18	Female	45–54	Disability rights org
P11	Medical doctor	9	Female	30–39	Community clinic
P12	Nurse	16	Male	40–49	Rehabilitation centre

NGO, non-governmental organization.

**TABLE 2:** Themes and capability dimensions.

Theme	Core process	Primary capabilities enhanced	Conversion factors addressed
Facilitating emotional transformation	Moving families from crisis to adaptive capacity	Emotions, affiliation and practical reason	Professional communication skills, timing of support
Building system navigation competence	Developing family advocacy and coordination skills	Practical reason, control over environment	Information access, professional coordination
Creating inclusive participation opportunities	Enabling meaningful community and educational engagement	Play, affiliation, bodily health	Transportation, institutional attitudes
Strengthening family functioning	Enhancing whole-family resilience and capability	Life, bodily health, affiliation	Financial resources, social networks
Fostering adaptive identity development	Supporting identity evolution beyond disability focus	Emotions, play and practical reason	Community acceptance, expectation frameworks

### Theme 1: Facilitating emotional transformation

This theme illustrates how experts support families in emotional restructuring after a diagnosis, moving beyond traditional grief counselling to approaches that build capability and enhance emotional and social connections.

Participants described a sophisticated approach to emotional support that systematically builds family emotional capabilities. The process involves recognising that emotional adjustment is not linear but requires ongoing capability development support:

‘The initial shock period can last weeks or even months. We’ve developed a staged approach where we provide information gradually, allowing parents to absorb and process each piece.’ (P11)

‘What I’ve observed is that grief isn’t linear for these families. We use grief mapping techniques to help them understand that their feelings are normal and valid.’ (P8)

Social workers emphasised the importance of building emotional resilience capabilities that extend beyond immediate crisis management to long-term family functioning and adaptation.

### Theme 2: Building system navigation competence

Rather than simply connecting families to services, experts described developing family capabilities for ongoing system navigation and advocacy. This theme revealed sophisticated approaches to building practical reasoning capabilities that enable families to effectively engage with complex service systems.

Medical participants emphasised building family decision-making capabilities through information sharing and skill development:

‘I teach parents to ask the right questions during appointments. They need to understand their child’s condition, treatment options, and what to expect. An informed parent is an empowered parent.’ (P11)

Social workers described comprehensive tools and strategies that enhance family control over their environment:

‘I spend considerable time helping families understand their entitlements – disability grants, tax benefits, school accommodations. The system is complex, and many families miss out on support simply because they don’t know it exists.’ (P3)

### Theme 3: Creating inclusive participation opportunities

This theme revealed how experts address environmental barriers that constrain children’s and families’ affiliation, play and bodily health capabilities. The focus extends beyond individual interventions to systemic modifications that enable meaningful participation.

Transportation emerged as a critical conversion factor that significantly impacts family capabilities:

‘Transport remains our biggest barrier in rural areas. We’ve started community car-pooling networks where families support each other with transportation to therapy appointments and school events.’ (P4)

Non-Governmental Organization participants described systemic approaches to enhancing inclusive environments through capacity building and environmental modification:

‘We’ve developed training programs for teachers to help them understand different developmental disabilities. Our approach is collaborative – we don’t just advocate for the child, we build capacity within the school system.’ (P5)

### Theme 4: Strengthening family functioning

This theme captures holistic approaches that enhance multiple family member capabilities simultaneously, recognising capability interconnectedness within family systems. Experts described interventions that maintain and develop capabilities across all family members.

Participants described interventions that maintain parents’ practical reasoning and affiliation capabilities while caring for a child with developmental disabilities:

‘I see parents who have completely lost themselves in caregiving. We work on what I call “identity preservation” – helping them maintain interests, friendships, and goals beyond their child’s disability.’ (P9)

Nurses described comprehensive whole-family approaches that recognise the interconnected nature of family capabilities:

‘Our approach is holistic – we don’t just support the child; we support the entire family unit. We offer sibling support groups and parent empowerment programs.’ (P12)

### Theme 5: Fostering adaptive identity development

This theme revealed how experts support families in developing new capability frameworks that incorporate disability as one aspect of family identity rather than a defining limitation. The process involves challenging deficit-focused perspectives while building realistic and hopeful capability frameworks.

Medical professionals described approaches that maintain hope while building realistic capability frameworks:

‘Success looks different for every family. We help parents redefine their expectations while maintaining hope and optimism.’ (P5)

Participants challenged deficit-focused approaches by systematically highlighting existing capabilities and potential for development:

‘We challenge deficit-based thinking by highlighting abilities rather than disabilities. I work with families to create “strength inventories” that document everything their child CAN do.’ (P12)

## Discussion

### Key findings

This qualitative study explored participant perspectives on how interdisciplinary approaches can improve family capabilities while supporting children with developmental disabilities. The participants’ perspectives reveal three key mechanisms through which collaborative networks of professionals may transform family support systems: coordinated care as capability enhancement, emotional support as capability development and whole-family capability enhancement.

The five themes reflect participants’ views that effective interdisciplinary support requires professionals to function as ‘capability architects’ who systematically design interventions that build sustainable family capacities across multiple domains simultaneously. From the participants’ perspectives, this represents a fundamental shift from episodic service delivery towards sustained capability development partnerships.

### Coordinated care as capability enhancement

The emergence of coordinated care delivery as a central theme aligns with extensive research demonstrating that fragmented services significantly impair family functioning and child outcomes.^12,17,18^ However, the perspectives shared by participants in our findings extend beyond traditional coordination models by revealing how they perceive themselves as functioning as ‘capability facilitators’ who systematically address conversion factors that enable or constrain family capabilities.

Participants described a sophisticated understanding of how different interventions interact to either enhance or constrain family capabilities, suggesting a need for more integrated training approaches that emphasise systems thinking and capability theory.

### Emotional support as capability development

The sophisticated approach to emotional support revealed in our findings challenges traditional grief counselling models by emphasising capability development rather than adaptation to loss. The phased emotional support approach described by participants reflects what White et al.^19^ identify as ‘capability security’ – ensuring sustainable access to emotional processing capabilities rather than crisis intervention.

Our findings suggest that effective emotional support in developmental disability contexts requires an understanding of how emotional capabilities interact with other capability domains, particularly practical reasoning and affiliation. This integrated approach to emotional support represents a significant advancement over traditional counselling models that focus primarily on individual adaptation.

### Whole-family capability enhancement

Our findings reveal participants’ emphasis on systematic attention to whole-family capability development, recognising that individual family members’ capabilities function as conversion factors for others’ development. This insight extends beyond traditional family systems approaches by demonstrating specific mechanisms through which enhancing one family member’s capabilities creates cascading improvements throughout the family unit.

The emphasis on maintaining parental identity and capabilities while providing intensive child support represents what participants described as a sophisticated understanding of capability interdependence within family systems. This approach challenges prevalent models that focus primarily on child outcomes without adequate attention to broader family functioning.

### Implications and recommendations

These findings have significant implications for professional training and service organisations. Based on the perspectives shared by participants, the capability-focused approach requires professionals to understand not only their disciplinary contributions but also how these interact with other domains to enhance or constrain family functioning. This suggests a need for interprofessional education emphasising capability theory, systems thinking and collaborative practice skills.

Drawing from participants’ experiences, the following practical recommendations emerge for implementation at the care management level:

- Establish integrated assessment protocols: Develop multidisciplinary assessment tools that evaluate family capabilities across emotional, practical reasoning, affiliation and environmental control domains, rather than focusing solely on child deficits. Implement these at initial intake and regular intervals to track capability development over time.
- Create family navigation support systems: Assign capability facilitators (trained case coordinators) to guide families through service systems, providing skill-building in advocacy, decision-making and resource access. These facilitators should maintain regular contact and actively teach families system navigation skills rather than simply making referrals.
- Implement phased emotional support pathways: Develop structured emotional support protocols that recognise the non-linear nature of adjustment, providing gradual information delivery, peer support connections and counselling resources matched to family readiness stages. Include regular emotional capability assessments to identify families requiring additional support.
- Build environmental modification action plans: For each family, create specific plans addressing key conversion factors (transportation, inclusive education access, community participation opportunities) with measurable milestones. Engage with schools, community organisations and transport providers to systematically reduce environmental barriers.
- Design whole-family intervention strategies: Develop care plans that explicitly address the capabilities of all family members, including siblings and parents. Establish sibling support groups, parental respite programmes and family identity development workshops as standard components of comprehensive care rather than optional add-ons.
- Establish interdisciplinary team coordination mechanisms: Create structured communication protocols (regular case conferences, shared digital care plans, designated team coordinators) to ensure all professionals working with a family understand the comprehensive capability development approach and avoid fragmented, siloed interventions.

Service organisations should consider restructuring support delivery to emphasise capability development over traditional service provision models. This includes developing assessment tools that evaluate capability enhancement rather than deficit reduction, implementing outcome measures that capture family capability development across multiple domains and establishing coordination mechanisms that support systematic capability enhancement approaches.

Policy implications include the need for funding models that support sustained capability development partnerships rather than episodic interventions, professional standards that emphasise capability enhancement competencies and service integration mechanisms that facilitate collaborative capability development approaches.

### Limitations

Several limitations should be considered when interpreting these findings. This study’s focus on expert perspectives limits our understanding of family experiences of capability enhancement; the findings reflect professional viewpoints rather than facts about family capability development. The study’s geographic limitation to one South African context suggests the need for cross-cultural validation of the capability enhancement framework. Additionally, the sample size of 12 participants, while appropriate for thematic saturation, may not capture all possible perspectives across the diverse range of professionals working in this field. The lead researcher’s role as both investigator and clinician working with families of children with disabilities introduced potential bias in data collection and interpretation. While reflexive journaling and peer debriefing were employed to address this, readers should interpret findings as expert perspectives shaped by specific professional contexts rather than universal truths. Furthermore, this qualitative study did not capture contradictory viewpoints or tensions between different professional approaches, which may exist but were not voiced by participants in the interview setting.

Future research should directly engage families and caregivers as co-researchers to validate and expand upon the professional perspectives documented in this study. Comparative studies examining alignment between professional perspectives and family experiences would strengthen understanding of capability enhancement processes, as would research exploring potential disagreements or contradictions among professionals about optimal approaches. Cross-cultural validation of the capability enhancement framework would strengthen its applicability across different contexts and service systems. Additionally, given the word limit of this article, future publications will provide more detailed analyses of individual themes and in-depth exploration of the mechanisms underlying capability enhancement.

## Conclusion

The findings demonstrate that from participants’ perspectives, effective interdisciplinary support for families of children with developmental disabilities requires professionals to function as ‘capability architects’ who systematically design interventions that build sustainable family capacities across multiple domains simultaneously. Participants described a fundamental shift from episodic service delivery towards sustained capability development partnerships.

The capability-focused approach articulated by participants offers a promising framework for transforming integrated care delivery by emphasising environmental modification alongside individual capability building, ultimately expanding family possibilities for achieving their full potential. The five themes of facilitating emotional transformation, building system navigation competence, creating inclusive participation opportunities, strengthening family functioning and fostering adaptive identity development provide a comprehensive framework for understanding and implementing collaborative approaches in developmental disability support.
